# Eculizumab in Shiga toxin-producing *Escherichia coli* hemolytic uremic syndrome: a systematic review

**DOI:** 10.1007/s00467-023-06216-1

**Published:** 2023-12-06

**Authors:** Paul L. de Zwart, Thomas F. Mueller, Giuseppina Spartà, Valerie A. Luyckx

**Affiliations:** 1 Department of Nephrology, University Children’s Hospital Zurich, Zurich, Switzerland; 2 Clinic of Nephrology, University Hospital Zurich, Zurich, Switzerland; 3 Epidemiology, Biostatistics and Prevention Institute, Department of Public and Global Health, University of Zurich, Zurich, Switzerland; 4 Brigham and Women’s Hospital, Renal Division, Harvard Medical School, Boston, MA USA; 5 Department of Paediatrics and Child Health, University of Cape Town, Cape Town, South Africa

**Keywords:** HUS, Hemolytic uremic syndrome, Typical HUS, Infection-associated, Eculizumab

## Abstract

**Background:**

Infection-associated hemolytic uremic syndrome (IA-HUS), most often due to infection with Shiga toxin-producing bacteria, mainly affects young children. It can be acutely life-threatening, as well as cause long-term kidney and neurological morbidity. Specific treatment with proven efficacy is lacking. Since activation of the alternative complement pathway occurs in HUS, the monoclonal C5 antibody eculizumab is often used off-label once complications, e.g., seizures, occur. Eculizumab is prohibitively expensive and carries risk of infection. Its utility in IA-HUS has not been systematically studied. This systematic review aims to present, summarize, and evaluate all currently available data regarding the effect of eculizumab administration on medium- to long-term outcomes (i.e., outcomes after the acute phase, with a permanent character) in IA-HUS.

**Methods:**

PubMed, Embase, and Web of Science were systematically searched for studies reporting the impact of eculizumab on medium- to long-term outcomes in IA-HUS. The final search occurred on March 2, 2022. Studies providing original data regarding medium- to long-term outcomes in at least 5 patients with IA-HUS, treated with at least one dose of eculizumab during the acute illness, were included. No other restrictions were imposed regarding patient population. Studies were excluded if data overlapped substantially with other studies, or if outcomes of IA-HUS patients were not reported separately. Study quality was assessed using the ROBINS-I tool for risk of bias in non-randomized studies of interventions. Data were analyzed descriptively.

**Results:**

A total of 2944 studies were identified. Of these, 14 studies including 386 eculizumab-treated patients met inclusion criteria. All studies were observational. Shiga toxin-producing *E. coli* (STEC) was identified as the infectious agent in 381 of 386 patients (98.7%), effectively limiting the interpretation of the data to STEC-HUS patients. Pooling of data across studies was not possible. No study reported a statistically significant positive effect of eculizumab on any medium- to long-term outcome. Most studies were, however, subject to critical risk of bias due to confounding, as more severely ill patients received eculizumab. Three studies attempted to control for confounding through patient matching, although residual bias persisted due to matching limitations.

**Discussion:**

Current observational evidence does not permit any conclusion regarding the impact of eculizumab in IA-HUS given critical risk of bias. Results of randomized clinical trials are eagerly awaited, as new therapeutic strategies are urgently needed to prevent long-term morbidity in these severely ill patients.

**Systematic review registration number:**

OSF Registries, MSZY4, Registration DOI https://doi.org/10.17605/OSF.IO/MSZY4.

**Supplementary Information:**

The online version contains supplementary material available at 10.1007/s00467-023-06216-1.

## Introduction

Hemolytic uremic syndrome (HUS), classically defined by the triad of microangiopathic hemolytic anemia, thrombocytopenia, and acute kidney injury (AKI), is a heterogeneous group of diseases sharing thrombotic microangiopathy (TMA) as its common pathology [1]. Most commonly, HUS is induced by infection with a Shiga toxin-producing *Escherichia coli* (STEC) and preceded by bloody diarrhea. The incidence of STEC-HUS is around 2 in 100,000 in the overall population, but about triple that in children aged < 5 years [1], making STEC-HUS the leading cause of community-acquired AKI among young children [2]. STEC-HUS (often described as ‘typical HUS’) can be a severe disease, with a 3–5% mortality rate [3], 20% of patients having neurological involvement [4], and 40% requiring kidney replacement therapy in the acute phase [4].^1^

At 5-year follow-up after STEC-HUS, a substantial proportion of surviving children have persistent kidney (30%) or neurological (4%) sequelae, some severe, which will necessitate life-long medical and/or rehabilitative care [5]. Other, less common infectious causes of HUS include *Streptococcus pneumoniae* and H1N1/Influenza A [1].

A minority of HUS cases is associated with a variety of non-infectious triggers, including disorders of the complement system, which are often genetic in origin (complement-mediated HUS, CM-HUS, often also described as ‘atypical HUS’ or aHUS) [1]. Certain drugs, malignancy, transplantation, and pregnancy also rarely trigger HUS, predominantly in adults [5].

Beyond supportive care, which is common to all types of HUS, specific treatment depends on its cause. In CM-HUS, treatment has been revolutionized by the introduction of eculizumab (Soliris®, Alexion Pharmaceuticals, Cheshire, CT, USA), a humanized monoclonal IgG antibody that binds to the C5 complement protein and blocks its cleavage [6], attenuating and preventing injury due to production of the terminal complement complex [7]. It has been shown to lead to hematologic normalization and preservation of kidney function in the majority of CM-HUS patients in both adult and pediatric populations [8–11]. Eculizumab has become the standard of care for CM-HUS, replacing plasma exchange in most CM-HUS cases. Eculizumab is, however, one of the most expensive medications in the world; the estimated cost of €500,000 per patient (> 40 kg) per year [12] effectively limits its use to high-income settings [13].

There is currently no targeted therapy for IA-HUS. Antibiotic treatment for STEC is controversial, since it might induce expression and release of Shiga toxin [14, 15] and has not been found to improve outcomes [16]. Plasma exchange and immunoadsorption are sometimes used in severe cases but are not supported by current evidence for both STEC-HUS and pneumococcal-associated HUS [17].

Activation of complement by Shiga toxin has been shown in mice [18] as well as in STEC-HUS patients [19, 20] and is associated with a more severe clinical course [21, 22]. This observation led to the seemingly successful off-label use of eculizumab in 3 children with severe STEC-HUS who required hemodialysis and had neurological involvement [23]. Since the publication of this report, a substantial amount of observational data and case reports have been published regarding the use of eculizumab in IA-HUS. However, the absence of randomized clinical trials raises doubts about the true benefits of eculizumab in IA-HUS, which is very costly and carries the risk of adverse effects. Indeed, some experts advise against its use outside of the context of a clinical trial [24].

In light of this controversy, this systematic review aims to summarize and evaluate all currently available data regarding the impact of eculizumab administration on medium- to long-term outcomes in IA-HUS.

## Methods

This systematic review was performed according to the PRISMA criteria (Preferred Reporting Items for Systematic Reviews and Meta-Analyses) [25]. Online Resource 1 shows the full criteria checklist. The review was registered with the Open Science Framework (OSF, registration number MSZY4).

### Databases and search strategy

**Fig. 1:**
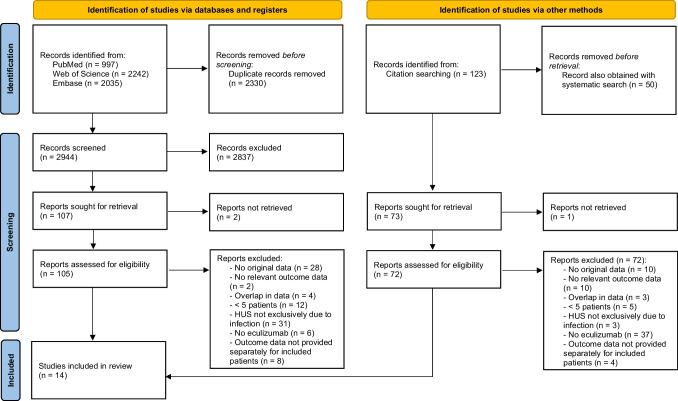
PRISMA flow diagram [25]

We searched PubMed, Embase, and Web of Science using a search strategy consisting of database keywords and text words as described in Online Resource 2. The final search occurred on March 2, 2022. The search terms comprised descriptions of hemolytic uremic syndrome or thrombotic microangiopathy, in combination with eculizumab, or variations of these terms. No filters were used. There were no language requirements. Gray literature (conference abstracts) was identified from Embase. Further references were identified through citation searching of identified articles (see Fig. 1).

### Selection criteria

Studies were included if original data regarding any medium- to long-term outcome was reported separately for IA-HUS patients who were treated with at least one dose of eculizumab at any stage during the acute phase of the illness. No additional requirements were placed on the patient population; in particular, patients with any age, comorbidity, or disease severity were included.

Medium- to long-term outcomes were defined as death or any indication of permanent damage to any organ, irrespective of when it was measured; there was no minimum follow-up time required, so that outcomes could include any measure of organ damage that persisted after the acute phase (and thus assumed to be chronic), or outcomes during the acute phase in case no follow-up data were available and there was reason to believe that this outcome represented permanent damage (e.g., stroke and surgical organ loss). Because of the expectation that studies would be variable in their reported outcomes, the medium- to long-term outcomes in this review were not limited to a predefined set. Instead, all outcomes that satisfy these criteria were included, most commonly death, kidney, and neurological outcomes. Studies were excluded if their data had substantial overlap with other included studies (i.e., multiple reports of the same patient cohort), if they included < 5 patients satisfying inclusion criteria, or if outcomes of included patients were not separately reported from those for non-included patients. Specifically, as no published randomized studies were identified, studies with lower-grade evidence (i.e., observational studies with or without control groups) were not excluded.

### Study selection, data extraction, and quality assessment

After duplicates were eliminated, studies were screened by one author (PdZ) for eligibility based on title, abstract, and, subsequently, full text. They were confirmed by a second author (VL). Discrepancies were resolved through discussion and consensus. Reference checks were performed for all included articles and for all other encountered articles for which a reference check seemed potentially relevant (mainly reviews on the topic).

Data from included studies were extracted into a data extraction form by one author (PdZ) and confirmed by a second author (VL). Extracted data included study design, patient recruitment period, type of study population, number of included patients, infectious agent, basic demographic information regarding age and sex, parameters used for estimating disease severity at admission and during the hospital stay, information regarding complement activation and genetic testing, indication for and dosage of eculizumab, time after disease onset at which it was administered, other treatments used, duration of follow-up, and patient outcomes. For the subset of studies which also reported outcomes in patients not treated with eculizumab, the same data was extracted for these patients if these otherwise satisfied the inclusion criteria.

Study quality for non-randomized studies was assessed independently by two authors (PdZ and VL) using ROBINS-I, a tool for assessing risk of bias in non-randomized studies of interventions [26]. Discrepancies were resolved through discussion and consensus. Studies where cases and controls were matched were evaluated in more detail to assess methodological quality, in particular regarding their choice of confounders used for the matching, fraction of patients not matched, covariate balance in the matched samples, and statistical method used to evaluate the treatment effect.

### Data analysis

Data was analyzed descriptively, stratified by study design (i.e., matching, inclusion of control group). No new statistical analysis or meta-analysis was performed, as the data are too heterogeneous and not of sufficient quality to permit merging.

Statistical significance of any difference in outcomes between eculizumab-treated and non-eculizumab-treated patients from the same study are reported on the study level, in cases where such data were available. These data are interpreted with caution, as in observational studies, these patient groups cannot be expected to be equivalent in terms of disease severity. When results were reported separately for multiple patient subgroups not treated with eculizumab, data about the subgroup most comparable (in terms of disease severity) to the eculizumab-treated patients were reported in this review. Whenever such data were available, data of a matched control group were used for comparative purposes.

## Results

In total, 2944 unduplicated studies were identified through the electronic database search (Fig. 1 shows the flow chart). After screening based on title and abstract, 107 studies remained, of which 2 were not retrievable; therefore, 105 underwent full-text eligibility assessment. Fourteen met the inclusion criteria [22, 27–39]. One study was only reported as a conference abstract [39]. Online Resource 3 provides an overview of why individual excluded studies were rejected. Reference checks led to another 72 full-text eligibility assessments, but ultimately did not lead to any additional included studies (Online Resource 3).

### Extracted data

Table 1 highlights the study population, study design, eculizumab indication(s), number of included patients, and outcomes of the included studies; full data is included in Online Resource 4. All included studies were observational, and no randomized controlled trials were identified. Eleven of 14 studies had a retrospective study design. Collectively, the studies report data from 386 patients with IA-HUS who received eculizumab. Nine studies also report data from patients not treated with eculizumab. Eleven studies focused on pediatric or young (< 25 years old) patient populations (*n* = 148). Overall, two-thirds of patients were female. STEC was identified as the infectious agent in 381 of 386 patients (98.7%). Most studies only included patients with severe disease, such as neurological involvement.

**Table 1:** Selection of extracted data

Study	Study population	Study design	Indication(s) for eculizumab	N of patients^f^ with/without eculizumab	N of patients who died	N of patients with medium- to long-term kidney damage	N of patients with medium- to long-term neurological damage	Other relevant outcomes
Pape et al. 2015 [27]	Pediatric patients with STEC-HUS with neurological symptoms treated with eculizumab	R	Evident neurological symptoms in the presence of active HUS	11	1/11	Long-term dialysis in 0/10 survivors	Severe neurological dysfunction in 1/10 survivors (NFS); only subjective deficits in 1/10 survivors (reduced performance regarding concentration and speed of mental processing), no permanent damage in 8/10 survivors	None
				None				
Muff-Luett et al. 2021 [28]	Patients ≤ 25 years old with STEC-HUS or non-STEC infection-related TMA treated with eculizumab^a^	R	Neurologic impairment in 43.4%, risk of death in 13%, other less common indications are not mentioned	23	2/23	Dialysis in 3/21 survivors (all with STEC-HUS), eGFR median increase of 73.3 (86.4) at follow-up compared to initiation of eculizumab in STEC-HUS (non-STEC infectious HUS) patients	No information	None
				None				
Percheron et al. 2018 [29]	Pediatric patients with severe^b^ STEC-HUS or D + HUS treated with eculizumab	R	Neurologic involvement in 20/33, cardiac and neurologic involvement in 8/33, cardiac involvement in 2/33, digestive involvement in 3/33	33	4/33	Dialysis in 0/29 survivors, eGFR below 60 in 12/29, significant proteinuria or microalbuminuria in 9/29	Neurologic sequelae in 5/29 survivors (behavioral disorders, delayed acquisitions, hypotonia, extrapyramidal syndrome, spasticity, and dystonia)	Pancreatic involvement in 4/33
				None				
Costigan et al. 2022 [30]	Pediatric patients (≤ 16 years old) with STEC-HUS with neurological involvement^c^	R	‘Severe disease’ (not defined) combined with plasma exchange not being practical, not effective or overwhelming multi-system involvement	8	1/8^~^	No information^l^	Mild impairment in 1/7^~^ survivors (difficulty with complex motor tasks)	None
				14	0/14^~^	No information^l^	Mild impairment in 1/14^~^ (dysarthria, mild weakness)	None
Travert et al. 2021 [31]	Adult patients (≥ 18 years old) with STEC-HUS	R	Not explicitly mentioned; patients treated with eculizumab had more often dialysis, and more often stroke, coma, or convulsions	38	5/38^−^	Duration of dialysis > 90 days in 1/22^~m^	Neurological sequelae in 4/14^~m^ (NFS)	None
				58	14/58^−j^	Duration of dialysis > 90 days in 3/20^~m^	Neurological sequelae in 4/11^~m^ (NFS)	None
Monet-Didailler et al. 2020 [32]	Pediatric patients (< 15 years old) with STEC-HUS treated with eculizumab, plus a historical untreated control group	R	Severe acute neurological involvement in 10/18 (with cardiac involvement in 4 of them), increasing hemolysis requiring transfusions and the need for kidney replacement therapy in 5/18, severe acute pancreatitis in 2/18, treatment in the context of an outbreak without severe symptoms in 1/18	18	0/18^~^	Decreased eGFR in 5/18^−^, proteinuria in 3/18^−^, prevalence of high blood pressure requiring treatment 17%^−^	Neurological sequelae in 5/18^*^ (psychomotor delay with language and walking acquisition delay, strabismus, moderate motor impairment)	Other sequelae in 0/18
				36^ g^	0/36^~^	Decreased eGFR in 14/36^−^, proteinuria in 10/36^−^, prevalence of high blood pressure requiring treatment 11%^−^	Neurological sequelae in 1/36^*^ (psychomotor delay and attention deficit disorder)	Other sequelae in 1/36 (pancreatic pseudocysts)
Ağbaş et al. 2018 [33]	Pediatric patients with STEC-HUS from a single outbreak requiring kidney replacement therapy^c^	R	Multi-organ failure in 2/9^e^, severe hematologic involvement in 5/9^e^, prolonged anuria in 2/9^e^, neurological involvement in 1/9^e^, gastro-intestinal bleeding/involvement in 3/9^e^, AKI-anuria in 1/9^e^, suspicion of complement factor H mutation in 1/9^e^	8	1/8^~^	Proteinuria 4/5^−m^, eGFR 109^−m^, hypertension 1/5^−m^	Neurological sequelae in 0/5^~m^	None
				11	0/11^~^	Proteinuria 3/9^−m^, eGFR 81^−m^, hypertension 1/9^−m^	Neurological sequelae in 0/9^~m^	None
Kielstein et al. 2012 [34]	Adult patients with STEC-HUS from a single outbreak treated with therapeutic plasma exchange^c^	R	No information	193	5/193^−^	Dialysis requirement in 9/193^−^, median creatinine 1.4 mg/dL^*^	Severe neurological disorders in 5/193^−^ (seizures) (-)	None
				241	9/241^−j^	Dialysis requirement in 9/241^−^, median creatinine 1.2 mg/dL^*^ (*p* = 0.013)	Severe neurological disorders in 1/241^−^ (seizures)	None
Giordano et al. 2019 [35]	Pediatric patients with STEC-HUS with central nervous system involvement^c^	R	Severe CNS involvement	5	0/5^ k~^	Chronic kidney failure requiring a dialysis-transplant program in 1/5^~^	Neurologic sequelae in 1/5^~^ (NFS)	None
				7	0/7^~^	Chronic kidney failure requiring a dialysis-transplant program in 0/7^~^	Neurologic sequelae in 0/7^~^ (NFS)	None
Gitiaux et al. 2013 [36]	Pediatric patients with STEC-HUS with neurological impairment^d^ treated early (< 10 days) with eculizumab	P	Severity of the condition in 5/7 patients, ineffectiveness of initial plasma exchange in 2/7	7	2/7	Proteinuria in 3/5 survivors, mild kidney failure in 1/5	Neurological sequelae in 0/5 survivors	Persistent diabetes mellitus due to HUS-related pancreas injury in 1/7
				None				
Loos et al. 2017 [37]	Pediatric patients with STEC-HUS from a single outbreak	R	HUS, neurological symptoms and prolonged dialysis with slow recovery of kidney function	13 (of which 1 LTFU^h^)	1/13^ h~^	Kidney transplantation/dialysis in 2/11^~m^, median serum creatinine 0.70^−mn^, proteinuria in 3/10^−m^, hypertension in 4/11^−m^. In analyses not shown, eculizumab treatment was not associated with CKD stage ≥ 2	No information^l^	None
				77 (of which 16 LTFU)	0/77^~^	Kidney transplantation/dialysis in 0/61^~m^, median serum creatinine 0.66^−m^, proteinuria in 16/59^−m^, hypertension in 10/61^−m^. In analyses not shown, eculizumab treatment was not associated with CKD stage ≥ 2	No information^l^	None
Ullrich et al. 2013 [38]	Patients with diarrhea, positive stool testing for EHEC, and/or signs of HUS during a single outbreak treated with eculizumab^a^	P	Severe neurological complications refractory to standard plasma-separation	7	No information^l^	No information^l^	No information^l^	Benefit from eculizumab treatment regimen (not explicitly defined) in 0/7
				None^i^				
Netti et al. 2020 [22]	Pediatric patients (< 18 years old) with STEC-HUS with neurological involvement and tested C3 levels at admission^c^	R	Severe CNS involvement	10	1/10^ k~^	No information	No information	None
				9	0/9^~^			
Sellier-Leclerc et al. 2012 [39]	Pediatric patients with STEC-HUS	P	Not explicitly mentioned; patients treated with eculizumab were substantially more often in need of kidney replacement therapy	12	0/12^~^	Kidney failure in 0/12^~^	No information	None
				68	0/68^~^	Kidney failure in 0/68^~^		

*CKD* chronic kidney disease, *CNS* central nervous system, *D* + *HUS* diarrhea-associated HUS, *eGFR* estimated glomerular filtration rate, *EHEC* enterohemorrhagic *E. coli*, *HUS* hemolytic uremic syndrome, *LTFU* lost to follow-up, *NFS* not further specified, *P* prospective, *R* retrospective, *STEC* Shiga toxin-producing *E. coli*, *TMA* thrombotic microangiopathy

^a^We included a subset of the population that was included in the original study, as the complete patient population did not fulfill the inclusion criteria of this review; the included subset is mentioned here

^b^‘Severe’ is defined as acute kidney injury with eGFR < 35 in combination with acute pancreatitis or neurological manifestations or cardiac failure

^c^For patients not treated with eculizumab, we included only the subgroup that was most comparable to those who were treated with eculizumab. The description of the population applies to the subset of the population that was included in this review

^d^Defined as disturbances of consciousness, epileptic seizures, and focal neurological signs

^e^Data is available only for a larger group of patients, including either patients with other eculizumab treatment status or other patients who were not included in this review

^f^Includes only the patients included in this review. Upper row: patients treated with eculizumab; lower row: patients treated without eculizumab

^g^Patients from a historical control group

^h^One patient died in the acute phase and was therefore not originally incorporated in this study on intermediate outcomes

^i^Not including non-eculizumab patients, since no relevant outcome data are provided separately for non-eculizumab patients

^j^Next to this direct comparison, a separate matching analysis was performed, see Table 3

^k^These studies show partial overlap

^l^No information provided separately for the patient groups treated with and without eculizumab

^m^Follow-up data not available for all patients

^n^Includes only non-transplanted and non-dialysis patients

*Difference statistically significant

^−^Difference not statistically significant

^~^No statement regarding statistical significance of difference

Indications for eculizumab varied both within and across studies; in most studies, eculizumab was administered in patients with severe disease, for example severe kidney involvement (i.e., requirement of kidney replacement therapy), non-kidney organ involvement (most often but not limited to neurological involvement), and/or as rescue therapy with/after plasma exchange. In all 9 studies that included a non-eculizumab patient group, patients who received eculizumab were more severely ill than the patients who did not receive eculizumab. Kidney replacement therapy was necessary in the majority of eculizumab-treated patients in all studies that provided such information. Plasma exchange was also frequently used. Detailed study-level data on patient status at admission, eventual patient status, used treatments, and patient outcomes are outlined in Online Resource 4.

The timing of first eculizumab administration was variably described, either relative to symptom onset [34, 36], time of diagnosis of HUS [29, 32, 33, 37], or development of indication for eculizumab treatment [27, 30, 35], and was therefore not comparable across studies. In 4 studies, the median or average time from development of first symptoms or diagnosis of HUS to eculizumab administration was more than 10 days [33, 34, 36, 37]. In 2 studies, eculizumab was administered within 24 h after developing an indication [27, 30]. In 5 studies, timing of eculizumab administration was not described. Study-level data on eculizumab administration are provided in Online Resource 4.

### Quality assessment of studies

Using the ROBINS-I tool, risk of bias was assessed for each study across 7 different domains, yielding a judgement regarding overall risk of bias as shown in Table 2.

**Table 2:**
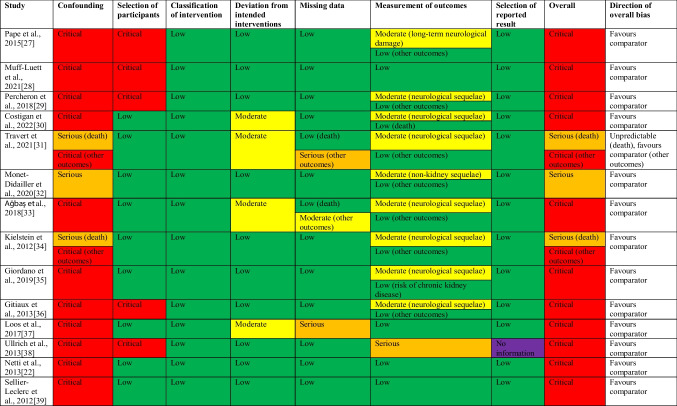
Risk of bias assessment using the ROBINS-I tool [26]

Risk of bias due to confounding had the most critical influence. It was judged to be critical in all studies that did not report outcomes in patients not treated with eculizumab [27–30, 36, 38], in those that did not attempt to control for confounding bias [22, 33, 35, 39], and in those that did not specify their analysis method [37].

Three studies used matching strategies to reduce confounding bias for at least some of their outcomes [31, 32, 34]. An analysis of the matching procedures and outcomes in these three studies is shown in Table 3. The matching was generally of low quality. The critical assumption (of strongly ignorable treatment assignment) on which the removal of confounding bias in matched studies rests is that the matched samples should be balanced regarding all covariates (both observed and unobserved) that are likely to be correlated with both the treatment assignment (the decision whether or not to treat the patient with eculizumab) and the health outcome of interest. Variables for which this is likely to hold, and which therefore should be matched for, include the health state of the patient prior to the decision of eculizumab treatment, such as laboratory values (Hb, thrombocytes, LDH, etc.) and complications indicating HUS-severity, as well as other factors such as age, comorbidity, which other treatments were used, and if these were successful or not. All three of the matching studies did not match for at least some of these important confounders, while none of the studies performed a sensitivity analysis for unobserved confounders and could therefore not exclude residual bias. Other limitations include a relatively high number of non-matched patients in two of the studies (i.e., it was only possible to match a proportion of patients within the study), as well as the use of tests to compare balance in baseline variables and to estimate eculizumab treatment effects on the outcomes which did not consider the matched nature of the selected sample by assuming independent sampling.

**Table 3:** Analysis of matching procedures and outcomes

Study	Travert et al. 2021 [31]	Monet-Didailler et al. 2020 [32]	Kielstein et al. 2012 [34]
Matching type	Propensity score matching	No information, most likely exact matching	Propensity score matching
Variables used for matching	Age, age-weighted Charlson comorbidity index, immune-deficiency, dialysis, stroke, coma or convulsions, TPE	Age, sex, presence of anuric acute kidney injury	Body weight, grade of neurologic symptoms, dialysis
Potential confounders not used for matching	Lab values, requirement of some other treatments than dialysis or TPE (i.e., ICU treatment, RBC transfusions etc.)	Lab values other than those indicating acute kidney injury, non-kidney complications, comorbidity, requirement of certain treatments (i.e., ICU treatment, RBC transfusions etc.)	Age, comorbidity, lab values, requirement of other treatments than dialysis or TPE (i.e., ICU treatment, RBC transfusions, etc.)
Formulation of propensity score model	Logistic model	Not applicable	Logistic model
Matching parameters	Nearest-neighbor method, caliper index equal to 0.3, 1:1 matching	1:2 matching, no additional information provided	1:1 matching with size of caliper adapted to achieve non-significant difference of covariates between the treatment and control groups
Propensity score overlap presented?	Yes	Not applicable	No
How much of the data was discarded due to non-matching?	8/38 patients from the treatment group with relatively high propensity scores were removed from the matching analysis	No discarded data	34/193 patients from the treatment group were removed from the matching analysis (no information provided about the reason)
Were treatment and control groups balanced according to the methods used in the respective study?	*Matching variables*: yes (no statistically significant differences between the matched groups) *Variables not used for matching*: no analysis performed	*Matching variables*: yes (no statistically significant differences between the matched groups) *Variables not used for matching*: no (patients in treatment group had significantly more neurological manifestations (55% (10/18) vs. 25% (9/36), *p* = 0.02)	*Matching variables*: yes (no statistically significant differences between the matched groups) *Variables not used for matching*: no analysis performed
Outcome variable(s)	Survival	*Kidney outcomes*: prevalence of decreased eGFR, proteinuria and high blood pressure requiring treatment at last follow-up *Non-kidney outcomes*: prevalence of non-kidney sequelae at > 1 year of follow-up	Survival
Conclusion after matching	*Survival*: no statistically significant difference between matched treatment and control groups (13.3% (4/30) vs. 23.3% (7/30) died, *p* = 0.51)	*Kidney outcomes*: no statistically significant difference between matched treatment and control groups (see Table 1 for the data) *Non-kidney outcomes*: statistically significantly higher prevalence of non-kidney sequelae in matched treatment group compared to control group (28% (5/18) vs. 6% (2/36), *p* = 0.02), reflecting the unbalance in the neurological presentation in the matched sample	*Survival*: no statistically significant difference between matched treatment and control groups (no further data provided)

*Ecu* eculizumab, *eGFR* estimated glomerular filtration rate, *ICU* intensive care unit, *RBC* red blood cell, *TPE* therapeutic plasma exchange

Because of the low matching quality, the potential reduction of confounding bias was limited in all 3 studies. Unbalanced treatment and control groups was confirmed in 1 study [32] (with significantly more initial neurological manifestations in the eculizumab than in the non-eculizumab group) and not tested in the other 2 studies. The remaining risk of confounding bias in these studies was therefore judged to be serious.

Since risk of confounding bias was at least serious in all studies, it dominated our judgement of overall risk of bias. Other domains of bias were less critical. Bias in selection of participants into the study was judged to be critical only in studies without non-eculizumab patients and low in other studies. Bias due to deviation from intended interventions was judged to be moderate in 4 studies with an imbalance in treatments other than eculizumab between the treatment and control groups, most notably the frequency of plasma exchange [30, 37], immunoadsorption [31], plasma therapy and other transfusions [33], and Protein C infusion [37]. Missing data was substantially unbalanced between treatment and control groups in one study [37]. Risk of bias in measurement of outcomes was generally low for outcomes of survival/death and kidney outcomes, as these are relatively objective. Neurological or other non-kidney outcomes, however, often required clinical judgement from clinicians who were generally not blinded to eculizumab treatment status. Risk of bias was judged to be moderate for studies regarding these outcomes. In one study [38], the outcome ‘clinical benefit of eculizumab’ was not defined and probably subjective, leading to serious risk of bias in this domain.

### Study outcomes

Study outcomes are summarized in Table 1 and Online Resource 4. Table 3 shows the outcomes of the three studies in which matching strategies were used in an attempt to reduce confounding bias.

#### Outcomes of studies only including patients who received eculizumab

Four studies [27–29, 36] reported outcomes only in patients who received eculizumab (Table 1). Overall, 9 of 74 patients died in the acute phase. Regarding medium- to long-term outcomes, dialysis was required in 3 of 65 survivors, chronic kidney disease [40] without need for dialysis was present in 13 of 34 patients with data [29, 36], and proteinuria or microalbuminuria persisted in 12 of 34 patients [29, 36]. Medium- to long-term neurological consequences were present in 7 of 44 survivors with data [27, 29, 36].

#### Outcomes of studies comparing eculizumab patients with non-eculizumab patients

##### Survival

None of the 9 studies reporting such data found an association between eculizumab use and survival in a direct analysis. In the two studies [31, 34] that found a lower death rate for patients treated with eculizumab, the difference was not statistically significant. In these studies, a matching analysis was performed to control for confounding bias. This, however, did not lead to a statistically significant difference in survival between eculizumab and non-eculizumab patients in either study (see Table 3).

##### Kidney outcomes

Kidney outcomes were variably reported and included creatinine, estimated glomerular filtration rate (eGFR), need for dialysis, proteinuria, or hypertension reported over the medium- to long-term. One study [34] reported a statistically significant difference in median creatinine at discharge with eculizumab-treated patients having a higher median creatinine at discharge than non-eculizumab patients (1.4 vs. 1.2 mg/dL, *p* = 0.013). In all other studies, including the only matching study providing kidney outcomes [32], kidney outcomes were not reported to be significantly different between treatment groups (see Tables 1 and 3).

##### Neurological outcomes

Neurological outcomes were also variably reported, with some studies providing detailed descriptions at the individual patient level, while other studies dichotomously classified patients as having (severe) neurological sequelae or not. One matching study [32] reported a statistically significantly higher prevalence of neurological sequelae in the eculizumab group compared to the control group at last follow-up (28% (5 of 18 treated patients) vs. 3% (1 of 36 untreated patients), *p* ≤ 0.02), possibly reflecting the baseline imbalance in the neurological presentation in the matched cohorts. All other (non-matching) studies did not report a statistically significant difference in neurological outcomes.

##### Other outcomes

Three studies [29, 32, 36] describe medium- to long-term pancreatic involvement in some HUS patients. However, no significant difference in pancreatic involvement between eculizumab- and non-eculizumab-treated patients is described. One other study [38] evaluated a composite outcome of ‘benefit of eculizumab treatment regimen’ in 7 patients and found no benefit.

#### ﻿Adverse effects

Three studies [28, 29, 33] reported bacterial or viral infection possibly related to eculizumab treatment in a total of 9 out of 64 patients, in one case leading to death [33], despite prophylactic administration of a meningococcal vaccine in all of these studies and antibiotics in at least two of three studies [29, 33]. Notably, bacterial infection was relatively frequent in the one study [28] that did not mention prophylactic antibiotic administration. Another study [32] reported late-onset, transient alopecia as a mild adverse event. All other studies report the absence of or do not mention any eculizumab-associated adverse events.

##### Complement activity

Various explorations regarding complement activity were performed in 4 studies to better understand the potential effect of eculizumab in IA-HUS. Using the CH50 assay to monitor complement blockade, one study [29] found evidence of more persistent blockade of complement activity in patients with more favorable compared to those with worse outcomes. Using the same CH50 assay, this finding was not duplicated in a second study [36], where 2 of 4 patients with a complete blockade of terminal complement activity died, whereas the 3 patients with partial blockade had relatively good outcomes. In two other studies, C3 levels at admission were analyzed. One study [33] found C3 levels to be similar between patients who would eventually receive compared to those who would not receive eculizumab. In contrast, the other study [22] found C3 levels to be significantly lower in those who would later receive eculizumab due to severe CNS involvement, indicating a more pronounced alternative pathway activation in these patients. The former study [33] did not find evidence of a beneficial effect of eculizumab, whereas the latter [22] states that eculizumab treatment was ‘effective’.

## Discussion

Overall, the outcomes for the eculizumab-treated patients included in this review are worse than those described in the literature of IA-HUS. For example, among the eculizumab-treated patients, the pooled mortality rate during the acute phase of the disease was around 6%, compared with around 3% in other studies describing (mostly) patients not treated with eculizumab [41, 42]. This observation likely reflects the general tendency among clinicians to restrict eculizumab treatment to patients with a severe disease course or as rescue therapy when all else has failed. Indeed, the seminal letter to the editor in 2011 which offered hope that eculizumab may be a solution for STEC-HUS also described that eculizumab was used ‘given the devastating prognosis’ [23].

Drawing any conclusion about the treatment effect of eculizumab in IA-HUS is severely hindered by the low quality of the available data. Fourteen studies were eligible for this systematic review, all were observational and judged to be at serious or critical risk of bias. Considering this limitation, eculizumab was not found to be associated with improved outcomes.

Given that this therapy tended to be given to the sickest patients, the potential for bias by indication is significant. Three studies did attempt to control for confounding through matching strategies and also found no evidence in favor of the eculizumab treatment. However, the risk of residual bias is substantial (see Table 3). If any positive treatment effect of eculizumab did occur, this may have been overshadowed by the poor prognosis of the patients who received it. Existing evidence in favor of the use of eculizumab in IA-HUS is currently limited to studies with ‘better than expected’ outcomes, but which lacked a control group [23, 27, 29, 35, 36, 39] or small studies and anecdotal case reports, for example the seminal report by Lapeyraque et al. [23].

It is possible that the treatment effect of eculizumab in IA-HUS was underestimated in most or all studies included in this review due to substantial residual bias in favor of the non-eculizumab patients. However, several other factors may also have contributed to the lack of an observed positive treatment effect. Firstly, most studies had a small sample size. Omitting the one outlier study with 193 eculizumab-treated patients [34], the average number of eculizumab-treated patients per study was less than 15. Secondly, eculizumab administration was generally delayed until patients developed severe disease or had not responded to other treatments (e.g., plasma exchange). It has been suggested that eculizumab may confer most benefit when administered early during the disease course [35]. This would be consistent with evidence that complement activation after STEC infection may resolve within a week [43, 44]. Eculizumab was administered to the majority of patients within a week after development of (certain) symptoms or HUS diagnosis in only 5 studies [27, 29, 30, 32, 35], whereas in others, the first dose of eculizumab was given after a median or average of over 10 days from symptom onset or diagnosis [33, 34, 36, 37]. Thirdly, the eculizumab effect may have been attenuated by the administration of complement components with plasma exchange or other plasma therapy in 11 studies [22, 29–34, 36–39]. Fourth, adverse events, in particular infectious ones, may have obscured any positive treatment effect of eculizumab’ although they were reported in just 9 patients and in most studies, prophylactic antibiotics were administered together with eculizumab.

It is of interest that indications for eculizumab in at least 5 of 14 studies [22, 27, 29, 35, 38] tended to be for non-kidney complications, especially neurologic deterioration, whereas isolated kidney failure or need for dialysis was reported by just 2 of 14 studies as an indication for eculizumab [32, 33]. This approach, which tends to ‘accept’ the need for dialysis as a given in this condition, and not as a severe complication that may also respond positively to ‘desperate measures’, can be questioned. The need for dialysis in AKI in anyone is not trivial and is associated with increased short- and long-term morbidity and mortality [45, 46]. In children especially, a life of kidney failure is complex and very challenging with all the compromises in quality and length of life associated with dialysis and transplantation.

This systematic review has several strengths. The review addresses an important clinical problem both from a disease severity point of view and from a resource utilization point of view. The literature search was systematic and comprehensive. To our knowledge, this is the first attempt to systematically analyze the impact of eculizumab therapy in IA-HUS. The restriction of included studies to those including at least 5 patients with eculizumab-treated IA-HUS was an attempt to reduce the impact of potential publication bias, since publication is more likely after successful than with unsuccessful use of eculizumab in studies with a small number of participants. Given the substantial risk of bias due to the observational nature of the included studies and the prevailing clinical use of eculizumab as rescue therapy in severe cases of IA-HUS, the use of the ROBINS-I tool to systematically evaluate bias is a significant strength here. Stratification of studies by matching and non-matching further attempted to minimize potential bias in interpretation of the findings.

This systematic review does however have several limitations, beyond the inherent high risk of bias in the included studies. Firstly, given the large heterogeneity among studies with respect to patient population, eculizumab indication, timing of eculizumab administration, utilization of other treatments, and reported outcomes, pooling of the data for a meta-analysis was not possible. Two included studies in particular [31, 34] had an adult patient population that deviated from the ‘classical’ pediatric patient population known to suffer from IA-HUS. The largest of these studies [34], providing half of the eculizumab-treated patients included in this review, focused on the 2011 German STEC outbreak. This outbreak had some unusual features (e.g., the STEC strain O104:H4 and age and sex of the patients), which therefore limits the generalization of its conclusion to other settings. However, similarly to the other included studies, these ‘unusual’ studies did not find an association between eculizumab administration and improved outcomes. Thus, their inclusion has not substantially impacted the conclusions of this review.

Secondly, almost all included studies report exclusively on STEC-HUS patients as studies reporting outcomes with other infectious causes were too small. Only 5 patients in this review had non-STEC IA-HUS. No conclusion can therefore be drawn regarding the treatment effect of eculizumab in non-STEC IA-HUS. Thirdly, not all studies reported or performed genetic testing to exclude the possibility of CM-HUS which may underlie a susceptibility to STEC-HUS and may have impacted treatment response. In STEC-HUS patients, there are often genetic and/or complement abnormalities [47]. Vice versa, many cases of CM-HUS are triggered by an infection, making the distinction in the acute phase often difficult. In genetic CM-HUS, eculizumab is known to be an evidence-based, effective treatment [1]. Thus, the treatment effect of eculizumab in IA-HUS can be overestimated if part of the treatment group is ‘contaminated’ with CM-HUS patients. On the other hand, the difficulty in distinguishing these two patient groups in the acute phase of the disease can make the case for empirical treatment of IA-HUS with eculizumab stronger.

In order to overcome the main limitations of the observational studies included in this review, two randomized clinical trials have been performed on the effect of eculizumab in STEC-HUS: ECULISHU, focusing mainly on kidney outcome, and ECUSTEC, focusing on general disease severity. The results of these studies were not available at the time of writing.

Despite the lack of conclusive data on the effect of eculizumab in IA-HUS, clinicians should not forget that there are multiple ‘conservative’ measures that may still optimize the chances for kidney and other organ recovery. These include optimization of volume status, blood pressure, nutrition, and minimization of nephrotoxin use. Peritoneal dialysis catheters may be placed in operating rooms. Anesthesiologists, surgeons, and intensivists should be aware to avoid significant hypotension during anesthesia or sedation. Intensivists together with nephrologists should establish clear blood pressure thresholds, both high and low, to optimize kidney perfusion and minimize hypoperfusion or hypertensive injury. Target blood pressures in children with AKI due to HUS are however unknown and should be studied. Avoidance of transfusions is important to reduce allo-sensitization in case kidney function does not recover fully given the risk of need for future transplantation. The risks and benefits of erythropoietin therapy while the disease is active requires further study [48]. Anecdotally, thrombocytosis may follow erythropoietin administration and further impact kidney perfusion. The use of antibiotics and analgesics requires careful thought, and doses should be adjusted appropriately. During dialysis, care should be taken to avoid precipitating hypovolemia, especially on automated peritoneal dialysis, and children should receive appropriate hydration if unable/unwilling to drink enough fluid. While not administering eculizumab may be justifiable based on the current lack of evidence of a positive treatment effect, it is important that adequate supportive management is not overlooked.

## Conclusion

The currently available observational evidence does not show a positive effect of eculizumab in the treatment of severe IA-HUS. However, given the high risk of bias, especially confounding bias, the treatment effect of eculizumab may be underestimated. A definitive conclusion can therefore not be reached. The results of randomized clinical trials are eagerly awaited, as new therapeutic strategies are urgently needed to prevent long-term morbidity in these severely ill, mostly young patients.

## Post-submission comment

After submission of this review, we became aware of the first results of the French Phase-3 randomized controlled trial (RCT) of eculizumab in children with STEC-HUS (ECULISHU), which were published online ahead of print [49].

In this trial, no statistically significant impact was found of eculizumab on the rate of kidney replacement therapy 48 h after first injection of eculizumab or placebo. Furthermore, eculizumab did not accelerate the resolution of TMA or reduce the incidence of non-kidney manifestations. However, the authors did find a statistically significantly lower proportion of patients experiencing kidney sequelae 1 year after study enrollment in the eculizumab group than in the placebo group (43.48% vs. 64.44%, respectively, *p* = 0.04). The authors conclude that eculizumab may reduce long-term kidney sequelae in this patient population. These results are not incorporated in the main body of this review but are of considerable interest, as these are the first published data in which confounding bias has been removed by randomization. In this trial, the patient population consists of patients with relatively mild disease. Patients with severe forms of STEC-HUS were excluded since many centers in France routinely prescribe eculizumab to children with severe STEC-HUS. Therefore, the investigators felt that including these children in the trial could have excluded some of them from potential benefit of compassionate use of eculizumab. In contrast, in the studies included in this systematic review, eculizumab was preferentially prescribed to severely ill patients; therefore, the patient populations in the RCT and those included in this review differ substantially. Clearly, larger prospective studies are still necessary to further determine the possible role of eculizumab in STEC-HUS patients, especially in those with severe disease, and especially as this therapy appears to have become integrated into routine care in some settings without strong evidence of benefit.

### Supplementary Information

Below is the link to the electronic supplementary material.

Supplementary file1 (PDF 170 KB)

Supplementary file2 (PDF 66 KB)

Supplementary file3 (XLSX 337 KB)

Supplementary file4 (XLSX 32 KB)

## Data Availability

All data generated or analyzed during this study are included in this published article (and its supplementary information files).

## Footnotes

1 Various definitions of HUS and its subcategories are in use in the scientific literature, which can be confusing. Some scholars define ‘typical HUS’ as STEC-HUS, and ‘aHUS’ as all other types of HUS, including non-STEC infectious HUS. Others define ‘aHUS’ as complement-mediated HUS. We use the term ‘HUS’ to describe types of TMA that primarily involve acute kidney injury. We prefer using causative terminology for subcategories, differentiating infection-associated HUS (IA-HUS), which includes STEC-HUS as well as HUS associated with other infections, from complement-mediated HUS (CM-HUS) and other types of HUS. Other types of TMA that are not primarily associated with kidney injury, such as thrombotic thrombocytopenic purpura (TTP), are not classified as HUS and not further considered in this article.
