# Supplementary material for: Eculizumab in Shiga toxin-producing Escherichia coli hemolytic uremic syndrome: a systematic review
Source: Pediatr Nephrol. 2023 Dec 6;39(5):1369–85. doi: 10.1007/s00467-023-06216-1 (PMC10943142; doi:10.1007/s00467-023-06216-1)
Supplement: Supplementary file 2 — Supplementary file2 (PDF 66 KB) [file 467_2023_6216_MOESM2_ESM.pdf]

Article title: Eculizumab in Shiga toxin-producing Escherichia coli hemolytic uremic syndrome: a systematic review

Journal name: Pediatric Nephrology

Author names: Paul L. de Zwart*, Thomas F. Mueller, Giuseppina Spartà, Valerie A. Luyckx

*Corresponding author, email paullambertus.dezwart@uzh.ch

Affiliation corresponding author: University Children’s Hospital Zurich, department of Nephrology, Zurich, Switzerland

All searches were performed on 2022, March 02

Pubmed:

((hemolytic uremic syndrome[MeSH]) OR (syndrome, hemolytic uremic[MeSH]) OR "hemolytic uremic syndrome"[tiab] OR "haemolytic uraemic syndrome"[tiab] OR HUS[tiab] OR "STEC-HUS"[tiab] OR "D+HUS"[tiab] OR (thrombotic microangiopathy[MeSH]) OR “thrombotic microangiopath*”[tiab] OR TMA[tiab]) AND (eculizumab[tiab] OR soliris*[tiab])

997 results

Web of Science (all databases):

TS=(“hemolytic uremic syndrome” OR “haemolytic uraemic syndrome” OR *HUS OR “thrombotic microangiopath*” or TMA) AND TS=(eculizumab OR soliris*)

2242 results

Embase:

('hemolytic uremic syndrome':ti,ab OR 'haemolytic uraemic syndrome':ti,ab OR hus:ti,ab OR 'stec-hus':ti,ab OR 'd+hus':ti,ab OR ‘thrombotic microangiopath*’:ti,ab OR tma:ti,ab) AND (eculizumab:ti,ab OR soliris*:ti,ab)

2035 results
